# Comparative analysis of curative effect of CT-guided stem cell transplantation and open surgical transplantation for sequelae of spinal cord injury

**DOI:** 10.1186/1479-5876-11-315

**Published:** 2013-12-20

**Authors:** Guanghui Dai, Xuebin Liu, Zan Zhang, Xiaodong Wang, Min Li, Hongbin Cheng, Rongrong Hua, Jing Shi, Renzhi Wang, Chuan Qin, Jianhua Gao, Yihua An

**Affiliations:** 1 Department of Cell Transplantation, General Hospital of Chinese people’s Armed Police Forces, Yongding Road, No. 69, Hai Dian District, Beijing 100039, China; 2 Department of Neurosurgery, Peking Union Medical College Hospital, Chinese Academy of Medical Sciences and Peking Union Medical College, Yongding Road, No. 69, Hai Dian District, Beijing 100730, PR China; 3 Department of CT Room Radiology, General Hospital of Chinese people’s Armed Police Forces, Yongding Road, No. 69, Hai Dian District, Beijing 100039, China

**Keywords:** Mesenchymal stem cells, Spinal cord injury, CT-guided puncture, Cell transplantation

## Abstract

**Background:**

This study compared the clinical efficacies, advantages and disadvantages of two transplantation approaches for treating spinal cord injury: open surgical exploration combined with local stem cell transplantation (referred to as open surgical transplantation) and local stem cell transplantation by CT-guided puncture (referred to as CT-guided transplantation).

**Methods:**

The patients were divided into the following three groups to perform a retrospective controlled study: Group A included nine patients who underwent open surgical transplantation, Group B included nine patients who underwent CT-guided transplantation, and Group C included nine patients who did not receive stem cell transplantation. The Abbreviated Injury Scale (AIS), the American Spinal Injury Association (ASIA) score and the motor evoked potentials (MEP) examination were utilized to compare the differences in the clinical efficacies. The advantages and disadvantages of the two transplantation approaches were also compared, including the surgical risks, the possibility of repeating the operation, the interval between surgery and rehabilitation exercises and the scope of conditions suitable for the operation.

**Results:**

Whether evaluated by the AIS grading scale, the ASIA score or the MEP results, there were significant differences in the clinical efficacy among the three patient groups. Group B exhibited the best clinical outcome, followed by Group A, and Group C fared the worst. The CT-guided transplantation had the advantages of lower surgical risk, the potential to repeat the operations within a short time-frame and a short interval between surgery and rehabilitation exercise compared with the open surgical transplantation. The conditions that are suitable for CT-guided transplantation versus the conditions suitable for open surgical transplantation are not identical. The application scopes for the two approaches had their respective strengths.

**Conclusions:**

CT-guided stem cell transplantation was confirmed as a safe and effective approach to treat sequelae of spinal cord injury with the advantages of simpler operation, minimal invasion, less adverse reaction and quicker recovery.

**Trial registration:**

Clinical trials registration number: ChiCTR-TNRC-12002477.

## Background

In both basic research and clinical applications, transplantations of mesenchymal stem cells (MSCs) derived from a variety of sources have been used to treat spinal cord injury (SCI) with positive results, as indicated by the efficacy and safety of this technique [1,2]. The current view is that the mechanism underlying stem cell (SC) treatments for SCI is associated with neuron replacement [3]; axonal regeneration and remyelination [4,5], neuronal protection [6-8]; promotion of vascular regeneration and improvement of the local blood supply [9-11]; induction of endogenous neural SC migration [12]; and regulation of the local inflammatory environment, systemic immune response and inflammatory response [13]. There are three commonly used cell transplantation approaches: local transplantation into the lesioned area [14], subarachnoid transplantation [15] and intravenous infusion [16]. Local transplantation into the lesioned area is the most commonly used technique and is considered the most effective approach [17] for treating SCI by SC transplantation. The classical procedure is open surgical exploration combined with local SC transplantation for SCI (referred to as open surgical transplantation), which involves exposing the injured spinal cord and the upper and lower edges of the normal spinal cord tissue by open surgery and performing intra-spinal cell transplantation under direct view [18]. This operation has the disadvantages of being high-risk and involving extensive trauma. There is a low potential to repeat the operation, and a long postoperative recovery is required before rehabilitation exercises can begin. To overcome these disadvantages, we developed a novel local SC transplantation technique for treating SCI. This method involves local SC transplantation by computed tomography (CT)-guided puncture for the treatment of SCI (referred to as CT-guided transplantation), and we compared these two methods herein.

## Methods

### Patients

Nine patients (8 male and 1 female) who previously received open surgical transplantation in our hospital were selected for this study. The patients’ mean (±SD) age was 36 ± 9.68 years, and their mean disease duration was 18.67 ± 7.68 months. Five cases were cervical SCIs, four cases were thoracic SCIs, six cases were complete injuries, and three cases were incomplete injuries. Preoperatively, six cases were identified as ASIA (American Spinal injury Association) Impairment Scale (AIS) grade A, one case was identified as grade B, and two cases were identified as grade C. Nine comparable cases from patients who received CT-guided transplantation and nine patients with SCI who did not receive SC transplantation were selected using age, duration of the disease, site of injury and degree of injury as the screening criteria. Together, these 27 patients made up the following groups: Group A patients received open surgical transplantation, Group B received CT-guided transplantation, and Group C received no SC transplantation. All the subjects in three groups were chronic SCI survivors and received decompression and internal fixation surgery at their acute phase of SCI. For Group A patients, the exclusion criteria included the following: (1) patients with inflammation or skin ulceration at the surgical site, (2) patients with a bleeding tendency or coagulation defects, (3) patients in poor general condition who could not tolerate surgery and (4) patients with systemic organ dysfunction who could not tolerate surgery. To minimize the influence of other factors that could also affect neurological rehabilitation during the assessment of the efficacy of SC transplantation, the following circumstances were also excluded from Group A: (1) patients who still showed recovery trends in terms of nerve function, (2) patients with adhesions at the injury site that required surgical intervention, (3) patients with significant compressive lesions at the injury site that required surgical intervention, (4) patients with progressive loss of nerve function for unknown reasons, (5) patients with associated syringomyelia who required surgical intervention and (6) patients with associated spinal cord or epidural vascular malformations that required surgical intervention. For Group B patients, the exclusion criteria included the following: (1) patients who still showed recovery trends in terms of nerve function, (2) patients with adhesions at the injury site that required surgical intervention, (3) patients with significant compressive lesions at the injury site that required surgical intervention, (4) patients with progressive loss of nerve function for unknown reasons, (5) patients with associated syringomyelia that required surgical intervention, (6) patients with associated spinal cord or epidural vascular malformations that required surgical intervention and (7) patients who could not be in the prone position or who could not remain in the prone position for more than 30 minutes. The selected patients did not differ in terms of rehabilitation exercises or other aspects of the treatment. The details of the patients are listed in Table 1. To avoid the influence of drugs on the neurological rehabilitation by nourishing the nerves and improving the microcirculation, the three groups of patients did not receive treatments with these drugs. To exclude the effect of rehabilitation exercises on neurological rehabilitation, the three groups of patients received formal rehabilitation exercises at the same hospital during the observation period. The study protocol was approved by the Committees of Ethics in Research of the General Hospital of Chinese People’s Armed Police Forces. All of the patients provided written informed consent and confirmed their willingness to receive UCMSC injection.

**Table 1:** Basic patient information

**Patient number**	**Sex**	**Age (Year)**	**Duration from injury to admission (Month)**	**Injury site**	**AIS grading**	
					**Before surgery**	**Six months after surgery**
A1	Male	28	14	C4-7	A	B
B1	Male	30	16	C4-6	A	B
C1	Male	25	13	C4-7	A	A
A2	Male	45	13	T8-10	B	C
B2	Male	47	17	T8-9	B	C
C2	Male	45	15	T8-9	B	B
A3	Male	30	16	T5-9	A	A
B3	Male	33	18	T7-8	A	A
C3	Male	29	16	T6-9	A	A
A4	Female	52	19	C4-5	A	B
B4	Female	51	23	C4-5	A	B
C4	Female	51	17	C3-5	A	A
A5	Male	24	14	C6-7	A	A
B5	Male	26	17	C5-6	A	B
C5	Male	27	15	C5-6	A	B
A6	Male	38	16	T1	A	A
B6	Male	35	19	T1-2	A	A
C6	Male	36	13	T1-2	A	A
A7	Male	26	21	C4-6	A	B
B7	Male	27	24	C4-7	A	B
C7	Male	26	18	C4-7	A	A
A8	Male	44	38	C5-6	B	B
B8	Male	46	36	C5-6	B	C
C8	Male	47	42	C5-6	B	B
A9	Male	37	17	T5-6	C	C
B9	Male	39	21	T4-6	C	C
C9	Male	36	14	T5	C	C

### Isolation and culture of UCMSC

**Figure 1:**
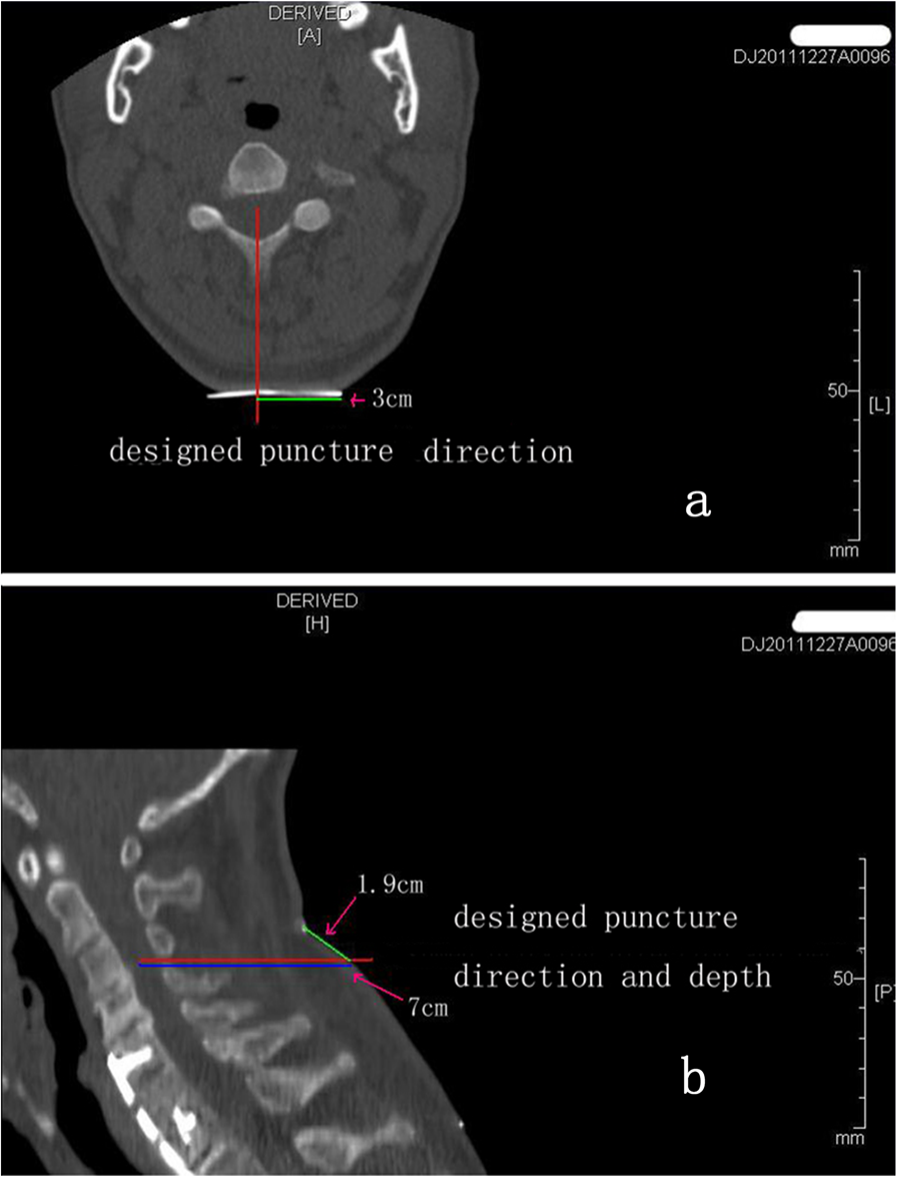
**CT machine ascertainment of the intended cell puncture site, direction and depth. a**, An axial (transverse) image. The red line indicates the upper intervertebral space of the intended puncture site and the direction. The green line shows that the intended puncture site is 3 cm away from the left edge of the marker. **b**, A sagittal image. The pink line represents the upper intervertebral space of the intended puncture site and the direction. The blue line shows the intended puncture depth of 7 cm, and the green line shows that the intended puncture site is located 1.9 cm below the upper marker.

Healthy pregnant women of normal gestational age who would undergo caesarean sections were selected. After obtaining signed written consent, an approximately 10 cm long section of the umbilical cord was removed during the surgery. The Wharton’s jelly was obtained after removing the surface amnion and blood vessels within the umbilical cord under sterile conditions, rinsed three times in normal saline, cut into small pieces with a size of approximately 0.5 cm3 and cultured in Dulbecco’s modified Eagle’s medium containing 10% fetal bovine serum. The residual tissue blocks were removed after 7–10 days. The cells that attached to the walls of the culture flask were trypsinized with 0.25% trypsin. The harvested cells were reseeded, and the medium was changed every 3 days. The cells were passaged every 5–7 days, then were frozen and stored in liquid nitrogen. Before being used for transplantation, the cells were thawed, cultured for 3 days. The flow cytometry was performed to detect the cell purity. All the percentages of CD105, CD90, CD73 and CD44 were higher than 95%, while none percentage of CD19, CD45, CD11b or CD34 were higher than 5%. Then the cells were used for transplantation. The results of flow cytometry were presented in Figure 1.

### Preparation of UCMSC

For the Group A and Group B patients, SCs were collected preoperatively, resuspended in normal saline to generate a cell suspension. For patients in Group A, 50 μl cell suspension (cell concentration was 8 × 10^5^ cells/μl) was injected into two sites of the spinal cord, 25 μl at each site. Totally 4 × 10^7^ cells were transplanted. For patients in Group B, 50 μl cell suspension (cell concentration was 4 × 10^5^ cells/μl) was injected into two sites of the spinal cord, 25 μl at each site. The transplantation was repeated once. Totally 4 × 10^7^ cells were transplanted.

### UCMSC transplantation

**Figure 2:**
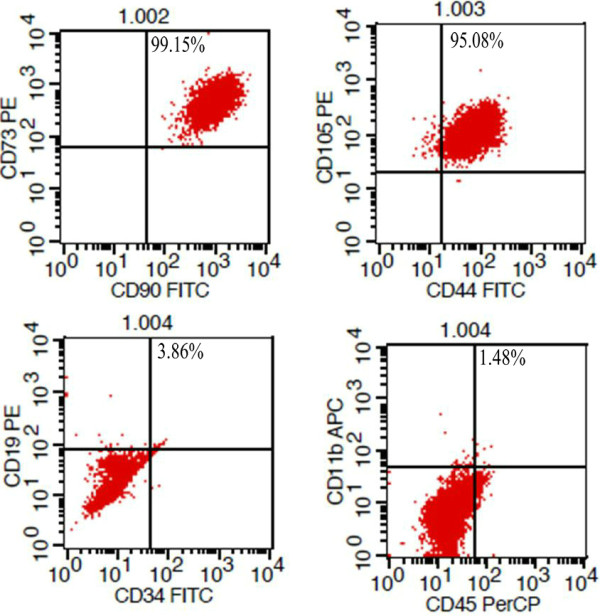
**Phenotype analysis of human UC-MSCs by flow cytometry assay.** All the percentages of CD105, CD90, CD73 and CD44 were higher than 95%, while none percentage of CD19, CD45, CD11b or CD34 were higher than 5%.

**Figure 3:**
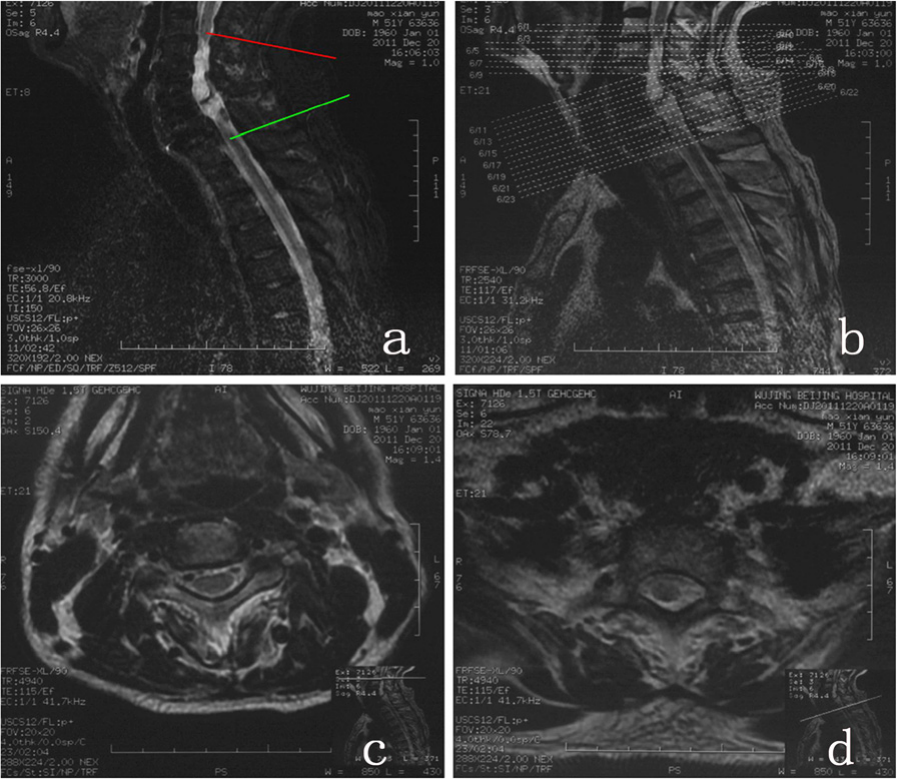
**Magnetic resonance imaging ascertainment of the local stem cell transplantation target by CT-guided puncture before operation. a**, Cervical MRI indicating that the patient had a C3-C6 injury; thus, the upper intervertebral space to be punctured was C3-C4, and the lower intervertebral space was C7-T1. The red line indicates the upper intervertebral space (C3-C4) to be punctured and the intended target for cell transplantation. The green line represents the upper intervertebral space (C7-T1) for the intended puncture and the intended cell transplantation site. **b**, **c**, **d**, The spinal cord conditions of the upper and lower targets designated for stem cell transplantation, indicating that the targeted sites in the spinal cord were healthy in appearance, contained no obvious lesions and were close to the injury site.

The patients in Group A received intravenous combined anesthesia in the prone position. The SCI site and the proximal and distal extensions of one vertebral body were completely exposed by the posterior midline approach, and the spinous process and lamina were removed carefully using rongeurs until the normal spinal cord was revealed. The dura was opened from the posterior midline under a microscope, and the prepared cell suspension was injected into both sides of the normal spinal cord near the injury site (the junction between the normal and abnormal spinal cord). The dura was then sutured tightly, and the wound was closed by conventional wound closure. After surgery, the patients were placed in the supine position, laid on hard wood plank beds, fixed to protect the surgical site and turned to the axial position for three weeks. The patients were turned over to prevent pressure ulcer occurrence and their legs were massaged to prevent venous thrombosis every 2 hours. Rehabilitation exercises were then initiated. The patients in Group B were kept in the prone position on the mobile bed of a CT machine, and the vital signs were monitored with a multifunctional monitor. The intervertebral space for the intended puncture was determined according to the site of the injury revealed by preoperative magnetic resonance imaging (MRI) and the sensory disturbance level of the patients determined by physical examination. The upper and lower ends of the normal spinal cord closest to the site of injury were used as the intended transplantation targets, and the intervertebral space for the intended puncture was further clarified based on the transplantation sites (Figure 2). The spiral CT scan parameters were 0.625 mm thickness, 120 kV and 50 mA. The intended puncture site, puncture direction and depth were determined based on the intervertebral space for the intended puncture, and the transplantation targets were based on the processing station of the CT machine (Figure 3). After surgery, the patients were kept in the prone position for six hours. Each patient received CT-guided transplantations twice, with an interval of at least 10 days. The rehabilitation exercises began on the first postoperative day after the full course of treatment was completed. None of the patients in Group C received any form of SC transplantation therapy and they were treated with rehabilitation exercises alone.

### Patient follow-up

The Group A and Group B patients were examined at our hospital six months after receiving SC transplantation. The Group C patients were examined in our hospital six months after enrollment.

### Evaluation of neurological function and electroneurophysiological examination

For the patients in Group A and B, evaluations of neurological function and electroneurophysiological examinations were performed prior to the SC transplantation and six months after completing the treatment. Neurological function was evaluated using the AIS grading scale and the ASIA standard developed by the ASIA [19]. The electroneurophysiological examination was conducted for the motor evoked potentials (MEP) evoked by the upper and lower limb movement. Group C patients received the neurological function evaluations and the electroneurophysiological examinations at enrollment and after a six-month interval. All the neurological evaluations were performed by two professional assessors who were blind to the study. All patients in three groups received same rehabilitation exercises in one hospital.

### Statistical analysis

The study data were analyzed using SPSS13.0 statistics software. The measurement data are expressed as the means ± standard deviations. The before and after scores for each group were compared using paired t-tests. The scores were compared among the three groups using one-way ANOVA. P < 0.05 was considered statistically significant.

## Results

Characterization of local stem cell transplantation by CT-guided puncture is presented in Supporting Information.

### AIS grading

In Group A, four cases exhibited changes in the AIS grade, and the improvement rate was 44.4%. Patients 1, 4 and 7 improved from grade A to grade B, Patient 2 improved from grade B to grade C and the other patients did not exhibit changes in the AIS grading. In Group B, six patients exhibited changes in the AIS grade, with an improvement rate of 66.7%. Patients 1, 4, 5 and 7 improved from grade A to grade B, Patients 2 and 8 improved from grade B to grade C and the other patients did not exhibit changes in the AIS grading. In Group C, one case exhibited a change in the AIS grade, with an improvement rate of 11.1%. Patient 5 improved from grade A to grade B, and the other patients did not exhibit changes in the AIS grading. There were significant differences in the changes in the AIS grading between the three groups of patients (P < 0.01), with Group B showing the most significant improvement, followed by Group A. Group C had the worst improvement rate (Table 1).

### ASIA score

In Group A and Group C, the preoperative motor score was 29.11 ± 22.55 and 29.11 ± 22.55, and the score was 31.3 ± 23.42 and 30.00 ± 23.28 at six months after operation, there was no significant difference compared to the baseline in both of two groups (P > 0.05). In Group B, the preoperative score was 28.67 ± 22.45 and the score six months after surgery was 36.44 ± 21.21. There was a significant difference between these two values (P = 0.008) (Table 2).

**Table 2:** ASIA scores of the three groups of patients at two time points

**Score subjects**	**Case number**	**Time**	**Group A**		**Group B**		**Group C**	
			**Mean ± Standard deviation**	**P value**	**Mean ± Standard deviation**	**P value**	**Mean ± Standard deviation**	**P value**
Motor	9	Before surgery	29.11 ± 22.55		28.67 ± 22.45		29.11 ± 22.55	
		After surgery	31.33 ± 23.42	0.149	36.44 ± 21.21	0.008	30.00 ± 23.28	0.052
Pain sensation	9	Before surgery	39.11 ± 27.08		38.00 ± 26.67		39.78 ± 26.86	
		After surgery	44.78 ± 26.78	0.006	53.33 ± 23.83	0.002	41.33 ± 26.55	0.111
Light touch sensation	9	Before surgery	40.22 ± 26.64		39.11 ± 27.15		40.22 ± 27.27	
		After surgery	48.22 ± 26.18	0.008	56.00 ± 23.07	0.004	42.11 ± 26.77	0.135
ASIA total score	9	Before surgery	108.44 ± 72.78		105.78 ± 72.86		109.11 ± 73.23	
		After surgery	124.33 ± 72.39	0.009	145.78 ± 63.06	0.002	113.44 ± 72.88	0.075

In Group A and Group B, the preoperative pain score was 39.11 ± 27.08 and 38.00 ± 26.67, the score six months after surgery was 44.78 ± 26.78 and 53.33 ± 23.83. There was a significant difference at pain score between pre-operation and 6 months (P = 0.006 in Group A; P = 0.002 in Group C). In Group C, the first score was 39.78 ± 26.86, and the score six months later was 41.33 ± 26.55. There was no significant difference between these two values (P = 0.11).

In Group A, the preoperative light touch score was 40.22 ± 26.64 and the score six months after surgery was 48.22 ± 26.18. There was a significant difference between these two values (P = 0.008). In Group B, the preoperative score was 39.11 ± 27.15 and the score six months after surgery was 56.00 ± 23.07. There was a significant difference between these two values (P = 0.004). In Group C, the first score was 40.22 ± 27.27, and the score six months later was 42.11 ± 26.77. There was no significant difference between these two values (P = 0.135).

In Group A, the preoperative ASIA total score was 108.44 ± 72.78 and the score six months after surgery was 124.33 ± 72.39. There was a significant difference between these two values (P = 0.009). In Group B, the preoperative score was 105.78 ± 72.86 and the score six months after surgery was 145.78 ± 63.06. There was a significant difference between these two values (P = 0.002). In Group C, the first score was 109.11 ± 73.23, and the score six months later was 113.44 ± 72.88. There was no significant difference between these two values (P = 0.075).

The assessment changes (delta value) of motor, pain, light touch and total scores of each individual from the baseline to the end of the follow-up were summarized in Table 3. The results showed that there were significant differences in motor, pain sensation, light touch sensation and ASIA total scores among Group A, B and C. Patients in Group B showed the most significant improvement, followed by patients in Group A. The patients in Group C showed the least improvement (Table 3).

**Table 3:** Assessment changes(delta value)in the ASIA scores over time among the three groups of patients

	**Motor**	**Pain**	**Light touch**	**Total score**
ΔA	2.22 ± 4.18 (ab)	5.67 ± 4.64 (ab)	8.00 ± 6.78 (ab)	15.89 ± 13.93 (ab)
ΔB	7.78 ± 6.63 (ab, bc)	15.33 ± 10.00 (ab, bc)	16.89 ± 12.64 (ab, bc)	40.00 ± 27.48 (ab, bc)
ΔC	0.89 ± 1.17 (bc)	1.56 ± 2.60 (bc)	1.89 ± 3.40 (bc)	4.33 ± 6.34 (bc)
P value	0.009	0.001	0.004	0.001

Note: ab indicates that the comparison of the improvements in the scores between Group A and Group B yielded P < 0.05; bc indicates that the comparison of the improvements in the scores between Group B and Group C yielded P < 0.05.

### MEP examination

In Group A, preoperative: All four limbs of Patients 1, 4, 5, 7 and 8 failed to elicit MEP waveforms. The limbs of Patient 9 generated recordable MEP, with the MEP of the upper limbs showing normal latency and amplitude and the MEP of the lower limbs showing delayed latency and low amplitude. The upper limbs of Patients 2, 3 and 6 all led to recordable MEP with normal latency and amplitude, but the lower limbs failed to elicit MEP waveforms. Six months after surgery: The right lower limb of Patient 2 elicited an MEP waveform. However, when compared with the normal waveform, it exhibited delayed latency and reduced amplitude. The right upper limb of Patient 8 elicited an MEP waveform. However, when compared with the normal waveform, it exhibited delayed latency and decreased amplitude. The MEP waveforms from both lower limbs of Patient 9 were improved, manifested as a reduced delay in the latency and increased amplitude. The other patients did not show significant improvement.

In Group B, preoperative: All four limbs of Patients 1, 4, 5, 7 and 8 failed to elicit MEP waveforms. All four limbs of Patient 9 could elicit an MEP, with the MEP of the upper limbs exhibiting normal latency and amplitude and the MEP of the lower limbs showing delayed latency and reduced amplitude. For Patients 2, 3, and 6, MEP with normal latency and amplitude were recorded from both upper limbs, whereas the lower limbs failed to elicit MEP waveforms. Six months after surgery: The right lower limb of Patient 2 elicited an MEP waveform. However, compared with the normal waveform, it showed delayed latency and reduced amplitude. The MEP from both lower limbs of Patient 9 showed some improvement, with shortened latency and increased amplitude, compared with the pre-treatment MEP. The other patients did not show significant improvement.

In Group C, first time: The four limbs of patients 1, 4, 5, 7 and 8 all failed to elicit MEP waveforms. MEP could be recorded from Patient 9, with the MEP from the upper limbs exhibiting normal latency and amplitude and the MEP from the lower limbs showing delayed latency and reduced amplitude. For Patients 2, 3 and 6, MEP with normal latency and amplitude could be recorded from the upper limbs, whereas the lower limbs failed to elicit MEP waveforms. Six months later: all patients failed to show significant improvement.

### Surgical risk

The intra-operative blood loss per patient in Group A was 230–360 ml, with an average of 192.22 ± 46.38 ml. The surgical duration was 150–180 minutes, with an average of 167.78 ± 10.03 minutes. There was neither pressure ulcer nor venous thrombosis occurrence in all post-operative patients. The Group A patients did not experience risk associated with X-ray radiation. The Group B patients did not experience intra-operative blood loss, and the operation duration was 25–50 minutes, with an average of 34.44 ± 8.08 min. The total dose of X-ray radiation received during a single surgery was 121.5-215.3 mGy, with an average of 164.12 ± 27.37 mGy. There were significant differences in the intra-operative blood loss, duration of surgery and the X-ray radiation dose received during the operation between the two groups (Table 4).

**Table 4:** Comparison of the surgical risks between Group A and Group B

	**Blood loss (ml)**	**Surgical duration (minutes)**	**Damage to spine stability**	**X-ray dose (mGy)**	**Anesthesia**	**Operation difficulty**	**Postsurgery adverse reaction (incidence %)**		
							**Fever**	**Headache dizziness**	**Neural radicular pain**
Group A	266.67 ± 70.1	241.11 ± 29.34	Yes	None	General	Difficult	22.2	22.2	11.1
Group B	None	34.44 ± 8.08	No	164.12 ± 27.37	Local	Easy	11.1	11.1	11.1

Attached figures:

### Adverse reactions

None of the Group A or Group B patients experienced complications, such as intracranial or extracranial infection and deteriorated symptoms. The two groups of patients were then compared in terms of the following adverse reactions. (1) Fever: In Group A, Patients 2 and 5 experienced fever, and the incidence was 22.2% (2/9); In Group B, Patient 4 had a fever, and the incidence was 11.1% (1/9). (2) Headache and dizziness: In Group A, Patients 1 and 6 experienced these symptoms, and the incidence was 22.2% (2/9); In Group B, Patient 4 experienced these symptoms, and the incidence was 11.1% (1/9). (3) Nerve radicular pain: In Group A, Patients 2 and 6 exhibited symptoms, and the incidence was 22.2% (2/9); in Group B, Patients 1 and 5 exhibited symptoms, and the incidence was 11.1% (1/9).

#### Postoperative recovery time before rehabilitation exercises

The Group A patients required a three-week postoperative fixing and protection time before initiating rehabilitation exercises; the Group B patients could begin rehabilitation exercises on the first day after surgery. The postoperative recovery time before rehabilitation exercises of the Group B patients was significantly shorter than for the Group A patients.

## Discussion

In this study, the two sets of AIS grades, ASIA scores and MEP results from the three groups of patients confirmed that local SC transplantation for SCI via either of two surgical techniques, CT-guided transplantation or open surgical transplantation, can effectively improve the neurologic dysfunction associated with SCI. The clinical outcomes in the two treated groups were significantly better than in the Group C patients, who did not receive SC transplants. In addition, the clinical efficacy in the Group B patients, who received CT-guided transplantation, was significantly better than in the Group A patients, who underwent open surgical transplantation. Furthermore, compared to open surgical transplantation, CT-guided transplantation was associated with significantly lower risks with respect to intra-operative blood loss, surgical duration, protection of the stability of the spine, anesthesia, operative difficulty and postoperative adverse reactions. Although CT-guided transplantation carries the X-ray-induced risk, the dose of X-ray radiation received by patients per treatment is far below the safety standard. We thus believe that the surgical risk of CT-guided transplantation is less than that of open surgical transplantation and that CT-guided transplantation is a safer SC transplantation approach. However, the two transplantation approaches each have their own advantages and disadvantages in terms of suitable and incompatible conditions. For the same conditions suitable for treatment by either approach, CT-guided transplantation can completely replace open surgical transplantation.

The conclusions of the clinical efficacy evaluation based either on the AIS grade or ASIA score were not consistent. The main reason is that the patients’ improvements in motor ability, pain sensation and light touch sensation were inconsistent, and the AIS grading scale is relatively general. Therefore, the ASIA scores can be used to individually evaluate the changes in the above three aspects. This study identified changes in the patients before and after transplantation, which are not necessarily revealed by the AIS grading. For example, Patient 8 in Group A was classified as grade B according to the AIS grading before SC transplantation. After treatment by SC transplantation, the patient’s pain and light touch scores were significantly improved. However, because the motor score did not exhibit significant improvement, the AIS grading of the patient did not change and remained grade B. Therefore, the ASIA score is more detailed and sensitive than the AIS grading for evaluating improvements in neurological function.

The MEP examination results suggested that the application of SC transplantation could improve neurological dysfunction in patients with SCI. The MEP is the electrical signal recorded in the distal spinal cord, peripheral nerve or muscles after stimulating the central nervous system. Therefore, MEP can directly reflect the functional status of the spinal cord descending fiber tracts and the peripheral motor nerves. The latency is the time frame between the stimulus initiation and a certain point of the response. The length of the latency is associated with the nerve fiber diameter and the size of the alpha motor fibers, which have the fastest conduction speed. The amplitude reflects the number of nerves measured and the degree of synchronized excitement, reflecting the total number of nerves and muscles involved in the implementation of the function. The MEP examination results showed that some patients regained MEP starting from zero, indicating that both the excitations leading to nerve impulses and the number of nerve fibers participating in the impulse were increased from zero. Some patients only exhibited shortened latency, whereas the amplitude showed no obvious improvement, suggesting that the conduction function of the spinal cord motor tracts was improved but that the number of fibers involved in nerve impulse conduction and the degree of excitement showed no significant improvement. The MEP findings do not fully agree with results from the AIS grade and ASIA score, which may be explained by the stimulation sites selected for the MEP examination. For example, when performing the upper limb MEP examination, the stimulation sites included the abductor muscle of the bilateral little fingers. However, the abductor muscle of the little finger is represented by the cervical spinal cord segment VII. If the injury site of the patient was located above cervical VII, the MEP waveform could not be elicited during the MEP examination. After SC transplantation therapy, if the relief of symptoms was manifested as a reduction in the sensory level or if the motor improvement was not sufficient to trigger an MEP, the MEP waveform would not be produced. Thus, under this circumstance, the MEP test results are not consistent with the AIS grade and the ASIA score.

Based on the AIS grading, ASIA scores and MEP results, both local SC transplantation approaches can effectively improve the neurologic dysfunction in patients with SCI. Of these approaches, CT-guided transplantation had the best efficacy, followed by open surgical transplantation; Group C showed no significant effects. To eliminate as many of the confounding factors that affect the analysis of the effects of SC transplantation, we excluded cases still showing a recovery trend in nerve function and cases with an unexplained decline in neurological function when screening for cases. Patients with adhesions at the injury site; obvious compressive lesions, syringomyelia or dural vascular malformations that required surgical intervention were also excluded. Therefore, the possibility of improving the neurological dysfunction by performing the corresponding surgeries for these diseases was minimized in this study. When designing this study, we ruled out the influences of drugs that nourish nerves or improve the microcirculation and of rehabilitation exercises. Therefore, the conclusion drawn from the results of this study that SC transplantation facilitates the rehabilitation of neurological functions for patients with SCI is reliable.

Based on the results, we conclude that CT-guided transplantation has many advantages compared with open surgical transplantation. (1) When comparing the intra-operative blood loss, duration of surgery and postoperative adverse reactions, CT-guided transplantation has distinct advantages as compared with open surgical transplantation. (2) Because open surgical transplantation requires opening the muscles, the spinous process and the lamina that stabilize spinal structure, this method can undermine the stability of the spine. In contrast, CT-guided transplantation does not change the anatomy of the structure that maintains the stability of the spine. It has obvious advantages in maintaining spinal stability. (3) CT-guided transplantation only requires a 9-gauge or 7-gauge puncture needle for the treatment. Because the diameters of the needles were only 0.9 mm and 0.7 mm, respectively, the surgical trauma is much less. The main difficulty in this operation is to adjust the puncture direction, but the operation is relatively simple. In contrast, open surgical transplantation requires the relevant knowledge and operation skills, including the anatomy of the spine, spine dynamics and structure, surgical incision, how to stop bleeding and microsurgery. It demands a wealth of basic knowledge and skilled surgical techniques. The difficulty of the surgery is significantly increased as compared with CT-guided SC transplantation. (4) CT-guided transplantation utilizes local anesthesia with very low anesthesia risk. In contrast, open surgical transplantation requires general anesthesia with higher anesthesia risks.

For CT-guided transplantation, the patients required multiple CT scans, thus exposing them to the risk of X-ray radiation, whereas the patients who received open surgical transplantation had no such risk. Therefore, it is necessary to assess the risks of the patients receiving X-ray radiation. Security requirements of the Association of the Heads of European Radiological Protection state that the accumulated amount of radiation received per person per year should not exceed 50 mSv, which is equivalent to 2,000 mGy, and that the accumulated amount of radiation received per person in 5 years should not exceed 100 mSv, which is equivalent to 4,000 mGy. During the CT-guided cell transplantation, we utilized low amp scans. For all nine patients, the dose of X-ray radiation received during each operation was 121.5-215.3 mGy, with an average of 164.12 ± 27.37 mGy. This amount is far below the safety standard developed by the Association of the Heads of European Radiological Protection. During the surgical operation, the doses of X-ray radiation to which the patients are exposed can be further reduced by decreasing the voltage and current during the CT scan and simultaneously increasing the spiral slice thickness. Thus, the dose of radiation received by patients undergoing CT-guided transplantation is within a safe range.

To the best of our knowledge, this is the first study to use CT-guided UCMSC transplantation in the treatment of spinal cord injury. However, some limitations of this study need to be considered. We only chose 9 patients in one group and 6 month time-points to explore the therapeutic effects of UCMSCs. More cases and follow-up time-points will be required to explore the safety and mechanisms of UMCSC transplantation. Thus, we cannot entirely exclude that some of the above-mentioned limitations may have influenced our results.

## Conclusions

CT-guided stem cell transplantation is a safe and effective approach to treat sequelae of spinal cord injury with the advantages of simpler operation, minimal invasion, less adverse reaction and quicker recovery. It can replace open surgical transplantation to a large extent.

## Abbreviations

UCMSCs: Umbilical cord-derived mesenchymal stem cells; SC: Stem cell; SCI: Spinal cord injury; AIS: Abbreviated Injury Scale; ASIA: American Spinal Injury Association; MEP: Motor evoked potentials.

## Competing interests

The authors declare that they have no competing interests.

## Authors’ contributions

GD, XL, ZZ, and YA contributed to the conception and design, the clinical treatment and manuscript writing. XW, HC and LM contributed to prepare the stem cells and performe laboratory experiments. RH and SJ contributed to the design, provision of study material, and data analysis. RW, QC, JG contributed to discussion and reviewed and edited the manuscript. All authors read and approved the final version of manuscript.
